# Epidermal Delivery of Retinyl Palmitate Loaded Transfersomes: Penetration and Biodistribution Studies

**DOI:** 10.3390/pharmaceutics12020112

**Published:** 2020-01-30

**Authors:** Eloy Pena-Rodríguez, Mari Carmen Moreno, Bárbara Blanco-Fernandez, Jordi González, Francisco Fernández-Campos

**Affiliations:** Topical & Oral development R+D Reig Jofre Laboratories, Gran Capitan Street 10, San Joan Despi, 08970 Barcelona, Spain; eloy.pena@reigjofre.com (E.P.-R.); maricarmen.moreno@reigjofre.com (M.C.M.); barbara.blanco.fernandez@gmail.com (B.B.-F.); jordi.gonzalez@reigjofre.com (J.G.)

**Keywords:** transfersomes, retinyl palmitate, biodistribution, skin, penetration, drug release

## Abstract

The alteration of retinoids levels in the skin can cause different disorders in the maturation of epithelial skin cells. Topical administration of these lipophilic molecules is a challenge that can be addressed by encapsulation into drug delivery systems. In this study, retinyl palmitate transferosomes formulated in cream were developed and the increases in the penetration of the active ingredients as well as the biodistribution were evaluated in vitro and in vivo. Transfersomes demonstrated a significant increase in the administration of retinyl palmitate to the epidermis by quantification of the active ingredients in the different layers of the skin, as well as by fluorescence microscopy of biopsies of non-dermatomized pig-ear skin. These results suggest that transfersomes may be an efficient vehicle for the delivery of retinoids to inner layers of the skin, such as the epidermis.

## 1. Introduction

Vitamin A derivatives are a group of lipid-soluble compounds including retinol, retinal, retinyl acetate, retinyl linoleate and retinyl palmitate (RP). Retinoids have important effects on skin cells. Retinoid levels in the skin are involved in the correct cellular maturation of keratinocytes, and when the skin is damaged, they induce keratinocyte proliferation and modulate epidermal differentiation [1]. Moreover, retinoids stimulate the production of extracellular matrix proteins such as collagen I by dermal fibroblasts. Alterations in these levels produce a de-structuring of corneocytes’ layers and, consequently, an increase in transepidermal water loss. Retinoids can also lighten hyperpigmented skin by decreasing melanocyte tyrosinase activity, inhibit the sebocyte proliferation and lipid synthesis, and alter their keratin expression. Alterations in retinoids skin levels can cause skin dehydration, lack of elasticity, sebum overproduction and hyperpigmentation, among other effects [2,3].

As retinoids cannot be synthesized by the body, they must be supplied through other sources [4]. Since these molecules are easily degraded by oxidation or photodegradation, and they are very hydrophobic, their topical bioavailability when applied on the skin surface remains quite low. In addition, retinoids have adverse effects such as hepatotoxicity, changes in lipid metabolism and bone density, teratogenicity, and they can cause significant skin irritation. Most of these effects occur after oral administration. Regarding topical administration, the main adverse effects are phototoxicity and skin irritation. One plausible mechanism of phototoxicity may be related to the formation of free radicals after the exposure of retinoids to UV light that damages the DNA. Relevant clinical studies or studies in animal models are therefore needed to establish whether the pro-oxidant activity of photoexcited vitamin A is observed in vivo, and to assess the related risks [5]. 

Skin irritation is linked to retinoids due to its pharmacological effects through retinoic acid receptor signaling [6]. Cytokines such as IL-1, TNF-α, IL-6, and IL-8 are thought to be more important in retinoid-induced dermatitis [7]. Retinyl Palmitate was irritating to rabbits’ skin, and a slight irritant to rabbit eyes. [4]. Thus, although retinoids have been classically incorporated into emulsions to overcome some of these limitations, skin irritation and photodegradability issues are still a problem [8]. The inclusion of retinol derivatives such as retinyl palmitate into nanocarriers for topical delivery is an interesting strategy that can lead to higher stability and enhance skin penetration [9]. Liposomes are a suitable choice for retinoids’ encapsulation, as the active ingredients can be incorporated into the membrane of the particles, ending in skin penetration and enhanced stability. 

Liposomes are spherical vesicles formed of a lipid bilayer and an aqueous cavity. They consist of phospholipids or synthetic amphiphilic molecules, usually combined with sterols to reduce their membrane permeability. Phospholipids tend to self-assemble in the presence of water due to their amphiphilic nature. The hydrophilic head is oriented towards the water, while the apolar tails are located in the inner part of the bilayer. The nature of the lipids will determine the liposomes’ properties. Saturated phospholipids can obtain liposomes with a lower permeability and greater stability than unsaturated phospholipids [10]. 

Classic liposomes usually accumulate in the stratum corneum and skin annexes. Therefore, they are not a good means to reach deeper layers of the skin or for transdermal absorption [11]. Therefore, different liposomal approaches have appeared. One example of these are ethosomes, which have ethanol in the vesicle cavity that behaves as a penetration promoter [12]. Another example are transfersomes, which are ultra-deformable liposomes [13]. This type of liposome has an “edge activator” (i.e., sodium cholate, sodium deoxycholate, span 80, Tween 80 or dipotassium glycyrrhizinate, among others) in its lipid membrane that allows it to increase its elasticity. There are several theories that explain the high penetration ability of these vesicles. The most accepted theory is that the high deformability of the transfersomes allows them to cross the intercellular channels of the stratum corneum [14,15]. Several researchers have demonstrated the improvement in topical penetration, for example, with retinol in dermatomized human skin and the keratinocites 3D model [9], and with lidocaine-loaded transfersomes, in order to avoid a painful local anesthetic injection [16]. 

The bilayer lipid matrix of cell membranes is composed of a mixture of different lipids. Among them, there is a growing interest in sphingolipids due their effects on skin cellular processes. Ceramides are a structurally heterogeneous and complex group of sphingolipids. It is well known that ceramides play an essential role in structuring and maintaining the water permeability barrier function of the skin. Ceramides maintain the dense crystalline structure of the lamellar lipids that are arranged between the corneocytes. They represent the 50% of lipids in the stratum corneum [17]. The rest of the lipids of the stratum corneum are cholesterol and free fatty acids. Together, they keep the lamellar structure of the stratum corneum and the barrier function of healthy skin in good condition. However, most skin disorders that have a diminished barrier function present a decrease in total ceramide content, with some differences in the ceramide pattern. Alterations in ceramide III levels are related to different skin diseases. In psoriatic skin, ceramides III and VII show a significant decrease versus normal stratum corneum [18]. In patients with atopic dermatitis, a decrease in the amount of ceramide III has been demonstrated to be correlated with an increase in transepidermal water loss [19]. Formulations containing lipids identical to those in skin and, in particular, ceramide supplementation, could improve disturbed skin conditions. Several authors have introduced ceramides in lipid-based vesicles to deliver them into the skin to restore lipid composition and to improve altered skin permeability [20,21]. Exogenously supplied, short-chain ceramides, such as ceramide III, induced keratinocyte differentiation in vitro and reinforced the pro-differentiation effects of other drugs [17].

Based on the effects of ceramide III and retinyl palmitate, as previously described, the formulations in this study were designed to improve and maintain the skin’s barrier properties. The aims of this work were the development and characterization of a ceramide III-based transfersome cream formulation encapsulating retinyl palmitate, and the study of RP biodistribution through the different skin layers. 

## 2. Materials and Methods

### 2.1. Materials

Ceramide III (Evonik Nutrition & Care, Essen, Germany), α-Tocopherol (Merck Chemicals and life, Barcelona, Spain), phosphatidylcholine (Lipoid, Ludwigshafen, Germany), Tween 80 (Croda Iberica S.A., Barcelona, Spain), Retynil Palmitate and Ethanol Absolute (Scharlab S.L. Barcelona, Spain) and purified water (Inhouse) were used to formulate the transfersomes. Dissodium EDTA (Sucesores de Jose Escuder, S.L., Barcelona, Spain), PEG-6 stearate (and) Ceteth-20 (and) Steareth-20 (Gattefosse España, Madrid, Spain), Cetyl Sterayl alcohol (Basf, Barcelona, Spain), medium chain triglicerides (Oximed expres S.A., Barcelona, Spain) and Xanthan gum (Azelis españa S.A., Barcelona, Spain) were used to formulate the emulsion. Metanol (Scharlab S.L., Barcelona, Spain), Nile Red, Hoeschst, phosphate buffer saline, paraformaldehyde (Sigma Aldrich, Madrid, Spain), uranyl acetate, optimal cutting temperature compound (IESMAT S.A., Barcelona, Spain), were used to perform the different analyses.

### 2.2. Production of Retinyl Palmitate-Loaded Transfersomes

Transfersomes were manufactured by the sonication method [22,23]. α-Tocopherol (0.02% *w*/*w*), ceramide III (0.10% *w*/*w*), phosphatydilcholine (1.78% *w*/*w*), tween 80 (0.10% *w*/*w*) and RP (1.10% *w*/*w*) were dissolved in ethanol (10% *w*/*w*) (organic phase). Then, milliQ water (qs 100% *w*/*w*) was added to the organic phase, and the system was vortexed for 1 min. Afterwards, the mixture was sonicated with a probe sonicator (amplitude of 80%, 5 min, Energy 7000 Ws, Frequency 23.88 kHz). The transfersomes’ suspension was left to settle at room temperature, protected from the light. Additionally, empty transfersomes (without RP) were fabricated, to study the effect of RP on the physio-chemical characteristics of the nanosystems. 

### 2.3. Transfersome Incorporation in a Cream Formulation

A mixture of surfactants (PEG-6 stearate (and) Ceteth-20 (and) Steareth-20 4% *w*/*w* and Cetyl Stearyl Alcohol 0.5% *w*/*w*), oils (Medium Chain Triglycerides or MCT 3% *w*/*w*) and an aqueous phase (12.6% *w*/*w*) with Xanthan gum (0.1% *w*/*w*) and disodium EDTA (0.1% *w*/*w*) were warmed up separately at 70–80 °C in a thermostatic bath. Once both phases were heated, the oil phase was added to the aqueous phase under mechanical stirring at 15,000 rpm for 2 min (Ultra-Turrax IKA T25, disperser unit S25KD 25F), and the mixture was allowed to cool down at 30 °C. Then, the transfersomes aqueous suspension, with RP at a concentration of 1.1% *w*/*w*, was added to the cream at a ratio of 1:1, so that the final RP concentration was 0.55% *w*/*w* (chosen taking the recommended concentrations for Retinol and Retinyl palmitate into account [5]). For the manufacture of the non-transfersomes emulsion, water was added up to 100%.

### 2.4. Transfersomes Physic-Chemical Characterization

RP-loaded transfersomes were subjected to a stability study in 25 ± 2 °C/60% ± 5% RH and 40 ± 2 °C/75% ± 5% RH chambers for 18 and 6 months, respectively, and conditioned in hermetically sealed glass vials.

Hydrodynamic size, polydispersion index (PDI) and zeta potential were studied through dynamic light scattering (DLS) using a Zetasizer Nano ZS (Malvern, UK). Dilutions of 1:10 in water were used for the measurements. 

Transfersome morphology was studied through transmission electron microscopy (TEM) using a Jeol JEM 1010 100 kv (Jeol, Tokyo, Japan). TEM grids were coated with formvar of a 1:10 transfersome dilution in milliQ water and incubated for 1 min at room temperature. Grids were then washed with water and stained with a 2% *w*/*w* uranyl acetate solution for 1 min at room temperature. Afterwards, they were dried in overnight and analyzed within two weeks of staining. 

The flexibility of the transfersomes was analyzed by extruding the transfersomes solution in an Avanti Mini Extruder with a 100 nm pore size polycarbonate membrane, at 1 mL of volume capacity. Pressure was applied by hand. The ability of the transfersomes to recover their initial size after extrusion was analyzed though DLS. The deformability index (DI) was defined as Equation (1),

(1) DI=(rprm)2

where *r_p_* is the radius of a the extruded transfersomes and *r_m_* is the radius of the membrane pores [24]. 

### 2.5. Cream Physic-Chemical Characterization

Appearance (visual observation), pH (pHmeter Crison Instruments S.A. Alella, Spain) and viscosity (Brookfield RDV-III Ultra, Spain. Spindler: SC4-21, Speed: 200 rpm, Temperature: 25 °C, Spain) were studied for the transfersome- and non-transfersome-loaded emulsions.

### 2.6. Diffusion Assay of RP-Loaded Transfersomes

In vitro diffusion of free RP and RP from the transfersomes (*n* = 6) was studied using vertical Franz Cells (VidraFoc, Spain, receptor compartment of 12 mL, diffusional area of 2.54 cm^2^). MCT was used as a receptor medium (RM) to keep sink conditions along the experiment. The dose of each formulation tested in the donor compartment was 240 mg (1.04 mg/cm^2^). The temperature of the experiment was maintained at 32 °C, and the stirring speed of the RM was 500 rpm. The membrane used was Polyvinylidne Fluoride (PVDF, Millipore, Spain) of a pore diameter 0.22 µm. 

Aliquots of 300 µL were taken at times 1, 2, 3, 4, 6, 24 and 30 h and injected into HPLC to quantify the amount of diffused RP.

### 2.7. RP HPLC Analysis and Encapsulation Efficiency 

The encapsulation efficiency (% EE) of RP in the liposomes was determined indirectly (Equation (2)). Briefly, transfersomes were centrifuged in 30 KDa Amicon ultracentrifugal filter (Merck Millipore) at 4500 rpm for 30 min. The amount of RP in the filtrate and in transfersomes were analyzed using a HPLC (Waters 2695, Spain), with a photodiode array detector (Waters 2996, Spain) with the corresponding calibration curve (Range 3.40 to 280 µg/mL with an r^2^ > 0.999). The column was a C18 (12.5 × 4.6 mm) with particle size of 5 µm. The mobile phase was an isocratic mixture of Metanol:water (98:2). The flow rate was 1.8 mL/min and the injection volume was 20 µL. the samples’ temperature was set at 5 °C and column temperature at 40 °C. % EE was determined using Equation (2),

(2) $$\%\;EE=\frac{W_{NE}-W_{T}}{W_{T}}\times 100$$

where *W_NE_* is the amount of RP quantified in the filtrate (RP not encapsulated) and *W_T_* is the RP quantified in the total amount of RP used for the preparation of transfersomes. 

### 2.8. Pig Skin Penetration Assays

Three to four month old male and female pigs were obtained from the Animal Facility at Bellvitge Campus of Barcelona University (Barcelona, Spain). Immediately after the animals (*n* = 3) were sacrificed, using an overdose of sodium thiopental anesthesia, the ears were surgically removed and frozen. On the day of experiment, ears were defrosted and the skin was excised.

#### 2.8.1. Skin Penetration Assay: Full Thickness Pig Ear Skin

Skin samples were mounted on Franz-cells (*n* = 6) according to the description in Section 2.6. The following formulations were tested: RP-loaded transfersomes, a free RP solution in MCT, transfersome-loaded emulsion (T emulsion) and a non-encapsulated RP emulsion. An amount of 240 mg of formula was administered in infinite doses in non-occluded conditions.

After 24 h, RP mass-balance was performed: RM was analyzed directly by mean HPLC, then the non-penetrated formulation in the donor compartment (non-penetrated) was recovered and RP was extracted from the emulsion according to the method described in Section 2.7. Skin pieces were taken and washed with distillated water (wash), and stratum corneum, epidermis and dermis were obtained, and RP extracted according to the methodology described in Section 2.8.2 and analyzed by HPLC [25,26]. 

#### 2.8.2. Skin Layers Recovery 

An incubation solution of RP in receptor medium at a concentration of 0.22 mg/mL was prepared, then skin layers were separated and incubated for 24 h with the incubation solution. After incubation, an extraction process was performed. Skin samples were subjected to 20 min of bath sonication in Metanol:water (98:2) and RP from the different skin layers was quantified by means of HPLC (Section 2.7). Stratum corneum was removed by applying 30 tape strips (pressure 225 g/cm^2^ for three seconds [27]). To separate epidermis and dermis, samples (after stripping) were immersed in 60 °C PBS for 2 min and excised with the help of forceps and a scalpel. The recovery percentage was applied to the results obtained in the penetration assays.

(3) % Recovery=QextractedSample mass(Q0h−Q24h)Sample mass×100

The percentage of recovery was calculated from Equation (3), where “Qextracted” is the amount of RP extracted from the sample after solvent incubation (Metanol:Water 98:2), “Q0h” is the initial amount of RP in the incubation solution at time 0, “Q24h” is the amount of RP in the incubation formula after 24 h of experiment and “Sample mass” is the mass of each skin layer sample. RP analyses were performed according to Section 2.7.

### 2.9. Fluorescence Biodistribution Assay

To study the biodistribution of the transfersomes in the skin, fluorescent-marked transfersomes were incubated on top of full-thickness ear pig skin samples using vertical Franz Cells (according to Section 2.8.1). Non-loaded transfersomes (autofluorescence control), Nile red-loaded transfersomes and free Nile red solution were added to the experiment (all at concentrations of 0.312 µg/mL).

After 24 h of incubation, skin samples were taken and cut into 0.25 cm^2^ pieces. They were then fixed with 4% paraformaldehyde (PF) for 30 min. Then, samples were washed with a phosphate buffer solution (PBS), and incubated in increasing concentrations of sucrose (up to 30% *w*/*w*) as a cryoprotectant. Samples were mounted in an optimal cutting temperature compound (OCT, from Fischer Scientific) and cut in the cryostat (Leica Biosystems) with a thickness between 30 and 50 µm, and placed on the superfrost slides with poly-lysine coating. 

The slides were washed with PBS to remove the remaining OCT and incubated with Hoeschst (2 µg/mL) for 30 min, and then washed with PBS. Samples were visualized by a Leica DMIRB Wide Field Fluorescence and Transmitted Light Microscope [28].

### 2.10. TEWL after In Vivo Topical Administration

An in vivo test was carried out in humans with T and NT emulsion. The study was conducted according to the Declaration of Helsinki. Volunteers gave their written consent. Transepidermal water loss (TEWL) was measured by the Vapometer (Delfin Technologies, Kuopio, Finland) before and after the application of the different creams to monitor SC removal [29]. A template with three application zones (NT emulsion, T emulsion and negative control: no emulsion applied) with an area of 2.54 cm^2^ for the forearm was used. 

Six male and female individuals (*n* = 6) with ages ranging from 23 to 44 years old participated. An amount of 0.025 g of each emulsion was applied on the skin by the same operator in the same conditions. After 2 h, skin was gently cleaned [30].

## 3. Results and Discussion

### 3.1. Transfersomes Physico-Chemical Characterization

The manufacture of empty liposomes (without RP) was unsuccessful. During the cooling process after sonication, the viscosity of the formula increased greatly, forming a gel, and no transfersomes were formed. 

The RP-loaded transfersomes obtained had a hydrodynamic diameter of 300.5 nm with PDI = 0.471, and a negative charge (Table 1). The % EE of RP was quantified by HPLC, as 100%. The RP limit of detection (LOD) and limit of quantification (LOQ) of the analytical method were 0.22 and 0.72 µg/mL, calculated by the signal to noise ratio). No RP was observed in the filtrate, demonstrating that all had been encapsulated. This fact, and the increased viscosity in empty transfersomes, seems to indicate that the entire RP is integrated with the transfersome membrane, and its presence is essential for the correct stabilization and formation of the nanosystems. 

**Figure 1:**
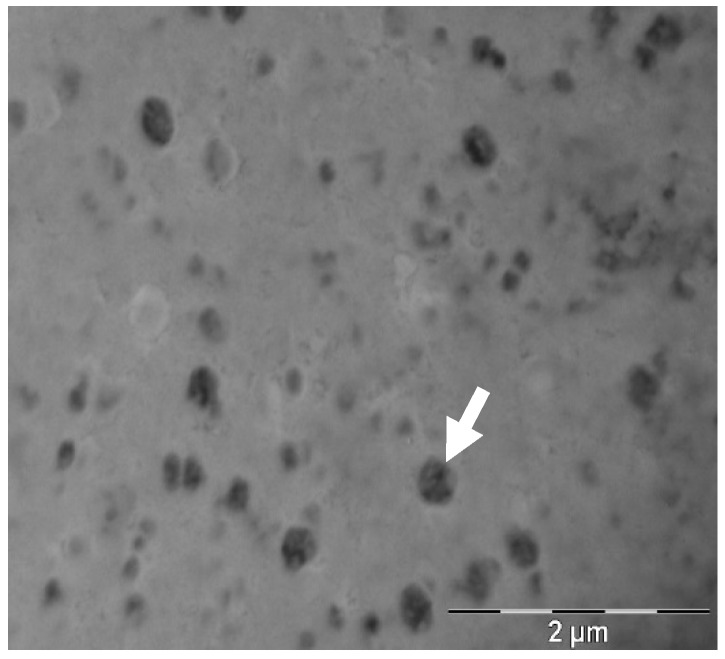
Transmission electron microscopy pictures of negative-stained transfersomes with uranyl acetate.

Transfersomes had a spherical shape (Figure 1). Mean particle diameter was also measured by TEM to corroborate the size results obtained in DLS with the TEM images, and to study the morphology of the vesicles. Using Image J software, the diameter obtained was 238.48 ± 29.74 nm. The fact that the diameter obtained in TEM is smaller than in DLS is due to the solvation of the transfersomes when measured in aqueous suspension.

**Figure 2:**
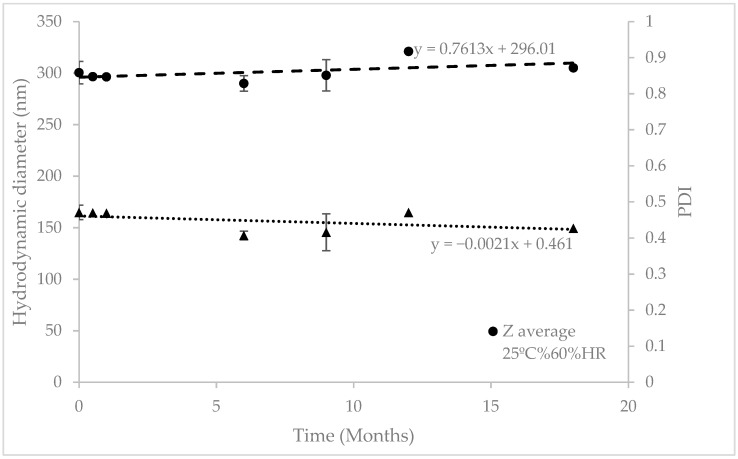
Stability studies at 25 ± 2 °C/60% ± 5% Relative Humidity (HR) over 18 months. RP-loaded transfersomes’ hydrodynamic diameter and polydispersity index, monitored over time.

**Figure 3:**
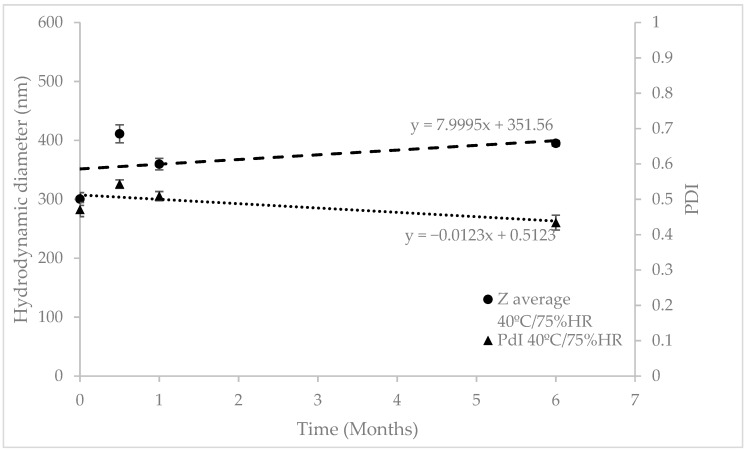
Stability studies at 40 ± 2 °C/75% ± 5% HR for six months. RP-loaded transfersomes’ hydrodynamic diameter and polydispersity index, monitored over time.

After manufacturing, transfersomes were stored in climate chambers at 25 °C/60% HR for 18 months and 40 °C/75% HR for six months (as accelerated conditions). Regression analysis of particle size and PDI was performed to check their evolution over time (Figure 2 and Figure 3). As can be seen in Table 2, the slope’s p-values were above 0.05, so the regression lines are statistically equal to zero, which means the RP-loaded transfersomes kept their hydrodynamic diameter and PDI stable at these conditions. The lack of significant variations at accelerated conditions, according to ICH Q1E [31], means the estimated product shelf-life can be extended to 36 months.

Transfersomes are defined as deformable liposomes, due to the presence of edge activators. To assess their flexibility, the Teixeira et al. method was used. This demonstrated the flexibility of their polymeric nanocapsules, based on the particles’ ability to recover their initial size after extrusion [32]. On the other hand, Yu-Kyoung Oh et al. [9] studied the deformability index of transfersomes manufactured with different edge activators. They obtained the highest DI with tween80 and tween20 (DI = 6 and 8.45, respectively).

In this study, RP transfersomes were extruded by a 100 nm pore size membrane, approximately three times smaller than their hydrodynamic diameter. The vesicle size and PDI before and after extrusion is shown in Table 3, as well as the DI. The difference in diameter is very small, showing their ability to recover their size after extrusion. However, a small proportion of the particles, probably the largest, are partially extruded, which explains the PDI decrease. These results are consistent with the results obtained by Teixeira et al. These results are also in agreement with Yu-Kyoung Oh et al., as the DI results are similar when choosing polysorbates as edge activators.

### 3.2. Transfersome Cream Physic-Chemical Characterization

Transfersome cream formulation and non-transfersome cream formulation appearance, pH and viscosities are shown in Table 4. The pH was in the same range as the human skin pH (between 4.5 and 5.5). Transfersome cream viscosity was higher, due to the increase in total lipid components. Both cream RP contents were assayed, and the obtained results were between 95% and 105%.

### 3.3. Synthetic Membrane RP Difussion Assay

Table 5 shows the % RP released at different timepoints from the transfersomes. Only 7.64% of RP was able to diffuse through the synthetic membrane after 30 h of experiment. Until six hours of experiment, no RP peak appeared in any of the chromatograms. As discussed previously, RP is completely integrated into the liposome membrane, forming a structural part of it. The presence of RP in the receptor medium at longer timepoints could be due to the drug diffusion from the liposomal lamella to the receptor medium (based on sink conditions), which is the classical release theory. However, although the membrane’s pore size does not allow transferome to pass through, it demonstrated their flexibility. Due to the lack of pressure from extrusion, vesicle translocation to RM across membrane pores takes a longer time to occur. Once transfersome crosses the pore, hydrophobic solution make them destructured and causes them to release RP. To ensure that intact transfersomes were able to cross the membrane pore, the experiment was replicated with water as the receptor medium instead of MCT, because DLS characterization would not be possible in this medium. After 24 h, an aliquot was extracted and measured by DLS. A population with a similar size (approximately 280 nm) appears, even though the count rate (kcps) was low (less than 200), indicating a low concentration of particles in the sample.

### 3.4. Franz-Cells Full-Thickness Pig Skin Penetration Assays

#### 3.4.1. Transfersomes’ RP Penetration

**Figure 4:**
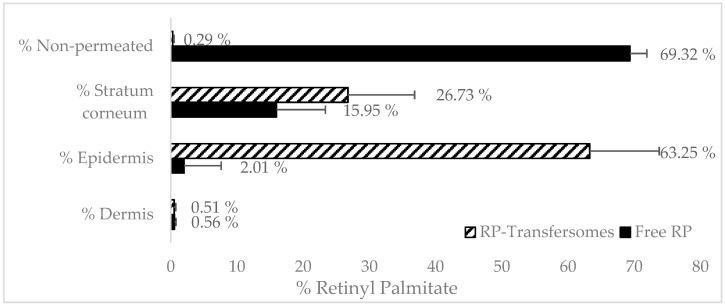
Black bar shows the free retinyl palmitate penetration. The black stripped bars show the RP penetration from transfersomes.

Figure 4 shows the penetration profile of RP from transfersomes and control (free RP solution in MCT) formulations in the different skin layers. Mass balance ranged between 90%–110% recovery in both cases. 

RP was not detected in the receptor medium in any of the formulations after 24 h, which seems to indicate that, after topical application, RP would not reach systemic circulation. 

Vehiculation in transfersomes has a significant effect on the biodistribution of RP in the different layers of the skin. In the case of non-vehiculated RP, most of the active ingredients did not penetrate the skin (69%) and only 2% reached the epidermis. The RP logP was around 15 [5] which is far from the optimal range (2–3) [33] to obtain maximum transdermal permeability. Its molecular weight is near 500 Da, which is considered the maximum value for transdermal absorption [34]. Therefore, it is expected to have a poor permeability. 

The percentage of RP found in the stratum corneum was higher in transfersomes (26%) than in free solution. Stratum corneum can act as a reservoir with a depot effect for epidermis absorption. However, part of this amount will also be eliminated during the natural desquamation cycle of the stratum corneum. 

RP found in epidermis was much higher in the case of transfersomes (63%), demonstrating that the increase in the delivery of RP into epidermis results in viable keratinocytes being found, which lead to its pharmacological effect. These results confirm the promoting effect of transfersomes for RP absorption, compared with a free solution of RP. Similar results were found by Yu-Kyoung Oh et al., who demonstrated an increase in the penetration of deformable liposomes based on the tween20 encapsulating retinol [9] compared with classic liposomes and free retinol solution. An increase in the epidermal delivery of RP loaded in vesicles was also obtained by Clares et al. [35]. In a study with dermatomized skin (0.4 μm) the liposomal systems demonstrates a greater penetration of nanoemulsions and solid lipid nanoparticles. Teixeira et al. [32] performed similar penetration assays on RP loaded in polymeric nanocapsules. Both of them obtained similar results to the ones shown in this research, of an approximately three-fold increase in RP levels in deep skin layers, compared with the amount found in the stratum corneum. 

#### 3.4.2. Emulsions RP Penetration 

It is very common to introduce liposomes or other topical drug delivery systems into emulsions to improve their attractiveness to final users. The inclusion of these formulations in emulsion could modify their physicochemical and biopharmaceutical characteristics. In order to evaluate the dilution effect and how the emulsification process could affect the transfersomes’ properties, a manufacturing simulation was carried out, because of the difficulty of finding transfersomes inside emulsions by electronic microscopy. RP transfersome solution was heated at the emulsion oil phase melting point (70–80 °C) for 15 min, then a water phase at the same temperature was added to the transfersome solution and mixed for 2 min with mechanical stirring at 15,000 rpm with the ultraturrax. Resulted solution was allowed to cold down and, after 24 h, vesicle size and PDI were measured by DLS, obtaining transfersomes of 266.4 nm and PDI 0.554. Even though this experiment cannot prove if the transfersomes fuse with the lipid components of the cream or not, it does demonstrate that transfersomes are able to resist the dilution effect and the emulsification process. 

**Figure 5:**
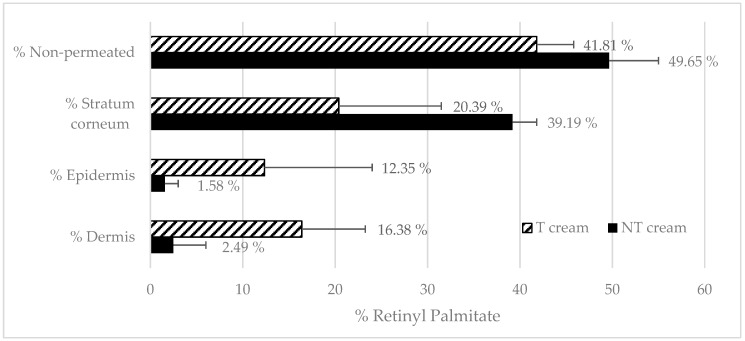
Black bar shows the RP penetration from Non-transfersome (NT) emulsion. The black stripped bars show the RP penetration from T emulsion.

Figure 5 shows the penetration of RP T emulsion and NT emulsion. It seems that the inclusion of transfersomes in an emulsion slows down the epidermis absorption, but, conversely, transfersomes increase the delivery of RP into epidermis. It seems that the emulsion could improve the dermis absorption of RT when transfersomes are used. In this case, the RP found in SC did not exhibit large differences between formulations. The presence of additional surfactants and emollients in the emulsion seems to improve RP disposition in SC when they are not loaded in transfersomes, but the epidermal and dermal delivery is still higher in the presence of transfersomes.

### 3.5. Fluorescence Biodistribution Assay

**Figure 6:**
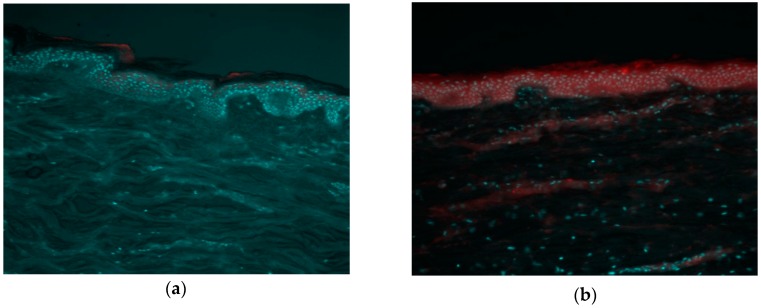
Fluorescence microscopy images of pig-ear skin cross-section. Red color corresponds to Nile red fluorescence and blue to Hoeschst staining of the cell nucleus. (**a**) Not vehiculized nile red control (image J, mean epidermis intensity 7846 ± 140 AU); (**b**) Nile red-marked transfersomes (image J, mean epidermis intensity 12,428 ± 254 AU). The images were captured using 10× magnifications

In order to confirm the increase in the epidermal delivery of RP in transfersomes, a fluorescence microscopy experiment was carried out with Nile red-loaded transfersomes and a free Nile red solution. Figure 6 shows the superposition of two fluorescence emission measurements from the same area of the skin sample (emission in the blue range for the cell nuclei stained by Hoeschst, and red for the Nile red formulation). As can be seen, the control of Nile red free solution (Figure 6a) only shows red fluorescence in the most superficial layer of the skin (stratum corneum), while the fluorescent marker loaded in transfersomes penetrates towards inner layers of the skin and, consequently, red fluorescence is observed (Figure 6b). These results agree with the results of the quantification of RP in the different layers of the skin, in which an accumulation of active ingredients in the epidermis and dermis was observed when it was vehiculized in transfersomes.

### 3.6. In Vivo Topical RP Penetration

The T and NT emulsions were tested in six volunteers for the screening of skin compatibility (based on TEWL measurement) and for RP quantification in the stratum corneum. Table 6 shows the TEWL (g/m^2^ h) measurements for each individual before and after application of the formulations. In order to study the stability of TEWL measurements, a *t*-test between the TEWL ratio and one was performed before and after application. No statistical differences were found, which means that the integrity of the skin is not affected by the formulations.

Two hours after application, the skin surface was cleaned, and tape strips were taken from each individual until TEWL values increased to 30–35 g/m^2^ h. A two sample *t*-test was performed to analyze the significance of the differences between the ratio and 1 before and after application, and there were no significative differences, as can be seen in Table 4 (*p* value 0.388).

## 4. Conclusions

Deformable transfersomes were successfully obtained with RP, integrated in the liposome membrane, with an estimated shelf-life of 36 months. RP-loaded transfersomes demonstrated an increase in penetration into the skin layers under the stratum corneum of the skin in vitro compared to a free RP control. Similarly, the inclusion of transfersomes to an emulsion increased RP skin penetration compared to the same emulsion without transfersomes. These results are reinforced thanks to the biodistribution experiment with fluorescence microscopy, where a significant increase in epidermis penetration was observed. The in vivo study demonstrated the compatibility of the tested formulations. Given these results, the developed transfersome formulation is a good candidate to increase the delivery of highly lipophilic drugs such as RP to the epidermis.

## Figures and Tables

**Table 1:** Transfersome physical chemical parameters, measured by dynamic light scattering (DLS) and HPLC. Assay (%) refers to the HPLC retinyl palmitate (RP) quantification assay respect to the nominal value of RP (1.1% *w*/*w*).

Sample	Hydrodynamic Diameter (nm)	PDI	Z-Potential (mv)	Assay (%)	EE (%)
Transfersomes	300.5 ± 10.9	0.471 ± 0.020	−9.48 ± 1.50	102.63 ± 0.51	100 ± 0

**Table 2:** Transfersome stability regression slopes.

Condition	25 °C/60% HR		40 °C/75% HR	
Response	Hydrodynamic Diameter	PDI	Hydrodynamic Diameter (nm)	PDI
Slope	0.761	−0.002	8.000	−0.012
*p*-value	0.231	0.277	0.546	0.275

**Table 3:** Transfersomes’ diameter, polydispersion index (PDI) and deformability index.

Sample	Hydrodynamic Diameter (nm)	PDI	Deformability Index
Transfersomes	300.5 ± 10.9	0.471 ± 0.020	8.12
Extruded Transfersomes	285.5 ± 9.7	0.247 ± 0.014	

**Table 4:** Physio-chemical parameters of transfersome and non-transfersome cream formulations.

Sample	Appearance	pH	Viscosity (cP)	Assay (%)
NT Cream	White-yellowish cream	4.86	64.70 ± 0.18	98.07 ± 0.68
T Cream	White-yellowish cream	4.81	100.15 ± 2.35	96.87 ± 0.72

**Table 5:** RP-released percentage through a synthetic membrane from a transfersome formulation.

Time (h)	Mean RP Released (%)	Standard Deviation (%)
4	0	0
6	0.36	0.57
24	6.81	6.19
30	7.64	6.61

**Table 6:** Transepidermal water loss (TEWL) average measures before and after the application of L and NT creams, as well as *p* value of the comparation between the ratio and 1 before and after application.

Time 0 h TEWL (g/m^2^ h)	Standard Deviation (g/m^2^ h)	Time 2 h after Application TWEL (g/m^2^ h)	Standard Deviation (g/m^2^ h)	Ratio TWEL 2 h/0 h	*p*-Value vs. 1
11.17	1.25	10.63	0.99	0.95	0.388

## References

[1] Gerard J., James G., Mezick A. (1993). Pharmacological effects of retinoids on skin cells. Ski. Pharm..

[2] Mukherjee S., Date A., Patravale V., Korting H.C., Roeder A., Weindl G. (2006). Retinoids in the treatment of skin aging: An overview of clinical efficacy and safety. Clin. Interv. Aging..

[3] Jean J., Soucy J., Roxane P. (2011). Effects of Retinoic Acid on Keratinocyte Proliferation and Differentiation in a Psoriatic Skin Model. Tissue Eng. Part A.

[4] O’Byrne S.M., Blaner W.S. (2013). Retinol and retinyl esters: Biochemistry and physiology. J. Lipid Res..

[5] Scientific Committee on Consumer Safety (2016). Opinion on Vitamin A (Retinol, Retinyl Acetate, Retinyl Palmitate).

[6] MacGregor J.L., Maibach H.I. (2002). The Specificity of Retinoid-Induced Irritation and Its Role in Clinical Efficacy. Exog. Dermatol..

[7] Kim B.H., Lee Y.S., Kang K.S. (2003). The mechanism of retinol-induced irritation and its application to anti-irritant development. Toxicol. Lett..

[8] Liu J.C., Wang J.C.T., Yusuf M., Yamamoto N., Kazama S., Stahl C.R., Holland J.P., Mather K., Aleles M.A., Hamada S. (1999). Topical Oil in Water Emulsions Containing Retinoids. U.S. Patent.

[9] Oh Y., Kim M.Y., Shin J., Kim T.W., Yun M., Yang S.J., Choi S.S., Jung W., Kim J.A., Choi H. (2006). Skin permeation of retinol in Tween 20-based deformable liposomes: In-vitro evaluation in human skin and keratinocyte models. J. Pharm. Pharmacol..

[10] Bozzuto G., Molinari A. (2015). Liposomes as nanomedical devices. Int. J. Nanomed..

[11] Braun-Falco O., Kortung H.C., Maibach H.I. (1992). Griesb Ach Conference: Liposomes Dermatics.

[12] Touitou E., Dayan N., Bergelson L., Godin B., Eliaz M. (2000). Ethosomes–novel vesicular carriers for enhanced delivery: Characterization and skin penetration properties. J. Control. Release.

[13] Jain S., Jain P., Umamaheshwari R.B., Jain N.K. (2003). Transfersomes—A Novel Vesicular Carrier for Enhanced Transdermal Delivery: Development, Characterization, and Performance Evaluation. Drug Dev. Ind. Pharm..

[14] Paul A., Cevc G., Bachhawat B.K. (1998). Transdermal immunization with an integral membrane component gap junction protein, by means of ultradeformable drug carriers, transfersomes. Vaccine.

[15] Cevc G. (1996). Transfersomes, liposomes and other lipid suspension on the skin, permeation enhancement, vesicles penetration and transdermal drug delivery. Crit. Rev. Ther. Drug Carr. Syst..

[16] Omar M.M., Hasan O.A., El Sisi A.M. (2019). Preparation and optimization of lidocaine transferosomal gel containing permeation enhancers: A promising approach for enhancement of skin permeation. Int. J. Nanomed..

[17] Coderch L., López O., de la Maza A., Parra J.L. (2003). Ceramides and skin function. Am. J. Clin. Dermatol..

[18] Motta S., Sesana S., Monti M., Giuliani A., Caputo R. (1994). Interlamellar lipid differences between normal and psoriatic stratum corneum. Acta Derm. Venereologica. Suppl..

[19] Matsumoto N.U.M. (1999). Difference in Ceramide Composition between “Dry” and “Normal” Skin in Patients with Atopic Dermatitis. Acta Derm. Venereol..

[20] Barbosa-Barros L., de la Maza A., López-Iglesias C., López O. (2008). Ceramide effects in the bicelle structure. Colloids Surf. A Physicochem. Eng. Asp..

[21] Shabbits J.A., Mayer L.D. (2003). Intracellular delivery of ceramide lipids via liposomes enhances apoptosis in vitro. Biochim. Biophys. Acta (BBA) Biomembr..

[22] Woodbury D.J., Richardson E.S., Grigg A.W., Welling R.D., Knudson B.H. (2006). Reducing Liposome Size with Ultrasound: Bimodal Size Distributions. J. Liposome Res..

[23] Jesorka A., Orwar O. (2008). Liposomes: Technologies and Analytical Applications. Annu. Rev. Anal. Chem..

[24] Cevc G., Gebauer D., Stieber J., Schätzlein A., Blume G. (1998). Ultraflexible vesicles, Transfersomes, have an extremely low pore penetration resistance and transport therapeutic amounts of insulin across the intact mammalian skin. Biochim. Et Biophys. Acta (BBA) Biomembr..

[25] European Centre for the Validation of Alternative Methods (CVAM) (2008). Test Guideline for Skin Absorption: In Vitro Method.

[26] Nangia A., Berner B., Maibach H.I., Bronaugh R.L., Maibach H.I. (1999). Transepidermal Water Loss Measurements for Assessing Skin Barrier Functions During in vitro Percutaneous Absortion Studies.

[27] Dabboue H., Builles N., Frouin E., Scott D., Ramos J., Marti-Mestres G. (2015). Assessing the Impact of Mechanical Damage on Full-Thickness Porcine and Human Skin Using an In Vitro Approach. BioMed Res. Int..

[28] Wilhelm K.P., Elsner P., Berardesca E., Maibach H.I. (1996). Bioengineering of the Skin: Skin Imaging and Analysis.

[29] Elmahjoubi E., Frum Y., Eccleston G.M., Wilkinson S.C., Meidan V.M. (2009). Transepidermal water loss for probing full-thickness skin barrier function: Correlation with tritiated water flux, sensitivity to punctures and diverse surfactant exposures. Toxicol. Vitr..

[30] Klang V., Schwarz J.C., Lenobel B., Nadj M., Auböck J., Wolzt M., Valenta C. (2012). In vitro vs. in vivo tape stripping: Validation of the porcine ear model and penetration assessment of novel sucrose stearate emulsions. Eur. J. Pharm. Biopharm..

[31] Baber N. (1994). International conference on harmorisation of technical requirements for registration of pharmaceutical for human use. Br. J. Clin. Pharmacol..

[32] Teixeira Z., Zanchetta B., Melo B.A., Oliveira L.L., Santana M.H., Paredes-Gamero E.J., Justo G.Z., Nader H.B., Guterres S.S., Durán N. (2010). Retinyl palmitate flexible polymeric nanocapsules: Characterization and permeation studies. Colloids Surf. B Biointerfaces.

[33] Benson H.A.E., Warkinson A.C. (2011). Transdermal and Topical Drug Delivery, Principles and Practice.

[34] Singh I., Morris A.P. (2011). Performance of transdermal therapeutic systems: Effects of biological factors. Int. J. Pharm. Investig..

[35] Clares B., Calpena A.C., Parra A., Abrego G., Alvarado H., Fangueiro J.F., Souto E.B. (2014). Nanoemulsions (NEs), liposomes (LPs) and solid lipid nanoparticles (SLNs) for retinyl palmitate: Effect on skin permeation. Int. J. Pharm..

